# Correction: Sequentially induced motor neurons from human fibroblasts facilitate locomotor recovery in a rodent spinal cord injury model

**DOI:** 10.7554/eLife.113550

**Published:** 2026-10-26

**Authors:** Hyunah Lee, Hye Yeong Lee, Byeong Eun Lee, Daniela Gerovska D, Soo Yong Park, Holm Zaehres, Marcos J Araúzo-Bravo, Jae-Ick Kim, Yoon Ha, Hans R Schöler, Jeong Beom Kim

**Keywords:** Human

Lee H, Lee HY, Lee BE, Gerovska D, Park SY, Zaehres H, Araúzo-Bravo MJ, Kim J-I, Ha Y, Schöler HR, Kim JB. 2020. Sequentially induced motor neurons from human fibroblasts facilitate locomotor recovery in a rodent spinal cord injury model. *eLife* **9**:e52069. doi: 10.7554/eLife.52069.

Published 23 June 2020

In the published article, there were errors in the metadata of the human fibroblast lines, as well as in the statistical values reported in Supplementary file 3.

To distinguish the two human fibroblast lines used in the study, we designated them as HF1 and HF2, using “HF” as an abbreviation for “human fibroblast”, rather than referring to the cells by their original cell line names.

Regarding HF1, the original cell line name is F134. We initially understood F134 to be an adult fibroblast line. However, we confirmed that F134 is a human fetal liver fibroblast line upon subsequent review of the relevant source publications.

Regarding HF2, data for this cell line were added during revision, but its source and catalogue number were inadvertently omitted. We acknowledge this omission and the confusion it may have caused. HF2 corresponds to a human adult dermal fibroblast line AG13004 (Coriell Institute for Medical Research).

In addition, the SD values in Supplementary file 3 were incorrectly entered during table preparation and have been corrected based on the original data.

**Corrected text in Results:**

To generate induced motor neurons (iMNs) from human fibroblasts,

**Originally published text in Results:**

To generate induced motor neurons (iMNs) from human adult fibroblasts (HF1),

**Corrected text in Materials and methods (Cell culture):**

Human fibroblast lines used in this study were HF1, a fetal liver fibroblast line (F134; Sgodda et al., 2013; Moslem et al., 2017), and HF2, an adult dermal fibroblast line (AG13004; Coriell Institute for Medical Research).

**Originally published text in Materials and methods (Cell culture):**

Human adult fibroblast lines, HF1 and HF2 were obtained from surgical resectates (Singhal et al., 2016), which have been obtained with the informed consent (Haridass et al., 2009).

**Corrected text in Key resources table:**

**Table:** 

Reagent type (species) or resource	Designation	Source or reference	Identifiers	Additional information
Cell line (*Homo sapiens*)	HF1	DOI: http://doi.org/10.2174/1566524011313050015. Sgodda et al., 2013		Parental cells
Cell line (*Homo sapiens*)	HF2	Coriell Institute for Medical Research	Cat.# AG13004	Parental cells

**Originally published text in Key resources table:**

**Table:** 

Reagent type (species) or resource	Designation	Source or reference	Identifiers	Additional information
Cell line (*Homo sapiens*)	HF1 (Female)	DOI: https://doi.org/10.4172/2157-7633.1000336 Singhal et al., 2016		Parental cells
Cell line (*Homo sapiens*)	HF2 (Male)	DOI: https://doi.org/10.4172/2157-7633.1000336. Singhal et al., 2016		Parental cells

**Corrected table (Supplementary file 3):**

Supplementary file 3. Summary of iMNIC induction from human fibroblast lines.

**Table:** 

Fibroblast line	Gender	Age	Cell type	Cells seeded per well	No. of colonies per well	Conversion efficiency to iMNICs	No. of established clones
HF1 (F134)	NA	NA	Fetal liver fibroblast	5,000	9.7±2.5	0.2 ± 0.05%	6
HF2 (AG13004)	Male	68	Adult dermal fibroblast	3,000	11.0±2.0	0.4 ± 0.07%	5

**Originally published table (Supplementary file 3):**

Supplementary file 3. Summary of iMNIC induction from human fibroblast lines.

**Table:** 

Fibroblast line	Gender	Age	Cell type	cells seeded per well	No. of colonies per well	Conversion efficiency to iMNICs	No. of established clones
HF1	Female	50	Adult dermal	5,000	9.7±1.6	0.2 ± 0.2%	6
HF2	Male	68	Adult dermal	3,000	11.0±1.4	0.4 ± 0.3%	5

**Inserted reference(s):**

Sgodda M, Möbus S, Hoepfner J, Sharma AD, Schambach A, Greber B, Ott M, Cantz T. 2013. Improved hepatic differentiation strategies for human induced pluripotent stem cells. Current Molecular Medicine 13:842–855. https://doi.org/10.2174/1566524011313050015

Moslem M, Eggenschwiler R, Wichmann C, Buhmann R, Cantz T, Henschler R. 2017. Kindlin-2 modulates the survival, differentiation, and migration of induced pluripotent cell-derived mesenchymal stromal cells. *Stem Cells International* 2017:7316354. https://doi.org/10.1155/2017/7316354

**Deleted reference(s):**

Singhal N, Arau´ zo-Bravo MJ Sinn M Zaehres H. 2016 . BAF complex enhances reprogramming of adult human fibroblasts. Journal of Stem Cell Research & Therapy 6:0336. DOI: https://doi.org/10.4172/2157-7633.1000336

Haridass D, Yuan Q, Becker PD, Cantz T, Iken M, Rothe M, Narain N, Bock M, Nörder M, Legrand N, Wedemeyer H, Weijer K, Spits H, Manns MP, Cai J, Deng H, Di Santo JP, Guzman CA, Ott M. 2009. Repopulation efficiencies of adult hepatocytes, fetal liver progenitor cells, and embryonic stem cell-derived hepatic cells in albumin-promoter-enhancer urokinase-type plasminogen activator mice. *The American Journal of Pathology* 175:1483–1,492. https://doi.org/10.2353/ajpath.2009.090117

The article has been corrected accordingly.

