# Supplementary material for: Sequentially induced motor neurons from human fibroblasts facilitate locomotor recovery in a rodent spinal cord injury model
Source: eLife. 2020 Jun 23;9:e52069. doi: 10.7554/eLife.52069 (PMC7311175; doi:10.7554/eLife.52069)
Supplement: Supplementary file 1. [file elife-52069-supp1.docx]

**Supplementary file 1. Primary antibodies used for ICC and IHC**

|  | **Antigen** | **Source** | **Isotype** | **Dilution** | **Localization** |
| --- | --- | --- | --- | --- | --- |
| ICC | NKX6.1 | DSHB | Mouse IgG1 | 1:200 | Nucleus |
|  | SOX2 | Santacruz | Goat IgG | 1:200 | Nucleus |
|  | PAX6 | DSHB | Mouse IgG1 | 1:100 | Nucleus |
|  | OLIG2 | Santacruz | Goat IgG | 1:200 | Nucleus |
|  | SV2 | DSHB | Mouse IgG1 | 1:100 | Cytoplasm |
|  | HB9 | DSHB | Mouse IgG1 | 1:100 | Nucleus |
|  | ISLT1 | DSHB | Mouse IgG2b | 1:100 | Nucleus |
|  | NCAM | DSHB | Mouse IgG2b | 1:200 | Cytoplasm |
|  | CHAT | Millipore | Rabbit IgG | 1:400 | Cytoplasm |
|  | TH | Millipore | Rabbit IgG | 1:200 | Cytoplasm |
|  | GFAP | Sigma | Mouse IgG1 | 1:500 | Cytoplasm |
|  | GFP | Abcam | Chicken IgY | 1:300 | GFP protein |
|  | MAP2 | Abcam | Mouse IgG1 | 1:400 | Cytoplasm |
|  | TUJ1 | Millipore | Mouse IgG1 | 1:500 | Cytoplasm |
| IHC | TUJ1 | Abcam | Rabbit IgG | 1:500 | Cytoplasm |
|  | MAP2 | Abcam | Mouse IgG1 | 1:400 | Cytoplasm |
|  | SV2 | DSHB | Mouse IgG1 | 1:100 | Cytoplasm |
|  | MBP | Millipore | Rat IgG2a | 1:100 | Myelin membrane |
|  | GFAP | DAKO | Rabbit IgG | 1:200 | Cytoplasm |
|  | GFP | Abcam | Chicken IgY | 1:300 | GFP protein |
