# Supplementary material for: Sequentially induced motor neurons from human fibroblasts facilitate locomotor recovery in a rodent spinal cord injury model
Source: eLife. 2020 Jun 23;9:e52069. doi: 10.7554/eLife.52069 (PMC7311175; doi:10.7554/eLife.52069)
Supplement: Supplementary file 2. [file elife-52069-supp2.docx]

**Supplementary file 2. Primers used for quantitative RT–PCT, RT–PCR and genomic PCR**

Quantitative RT–PCR primers for MN marker genes

| **Gene** | **Forward primer (5'-3')** | **Reverse primer (5'-3')** |
| --- | --- | --- |
| *HB9* | GCACCAGTTCAAGCTCAAC | GCTGCGTTTCCATTTCATCC |
| *ISL1* | GTTACCAGCCACCTTGGAAA | TGAATGTTCCTCATGCCTCA |
| *TUJ1* | GCAACTACGTGGGCGACT | CGAGGCACGTACTTGTGAGA |
| *LIM1* | TAGCAACGAGAATGACGACC | GGAACCAGACCTGAATGACG |
| *LHX3* | GACGCTGACCTAGGAGGA | CTGCACTTGAGACACTTGCT |
| *NKX6.1* | ATTCGTTGGGGATGACAGAG | CCGAGTCCTGCTTCTTCTTG |

Quantitative RT–PCR primers for HOX genes

| **Gene** | **Forward primer (5'-3')** | **Reverse primer (5'-3')** |
| --- | --- | --- |
| *HOXC4* | GGGTGAATTTCAGGGGAAATGAGG | CTCAAACTGAACAGCTCTGAGAGG |
| *HOXC5* | ATCAAGATCTGGTTCCAGAACCGC | AGGAAAAGCGCTTTTGTCTGTGGG |
| *HOXC6* | TTAGCACCGTCAGTGTTCCTATCC | TATACAGGAGGGTAACACGAAGGG |
| *HOXC8* | AGGAACCTGATGGAAACCTGAAGG | ATCAAACAGCGAAGGAGAGGAAGG |
| *HOXC9* | TAGAGTTAGTTCTACCCAGCGAGG | ACCTGGACCAAATACGATACAGGG |
| *HOXA7* | ACTACCTATTTTGTGCTGGCTGGC | GAGAAGGAGGGATTGATTCTAGGG |
| *HOXA9* | CAGGGTCTGGTGTTTTGTATAGGG | ACGCTTGACACTCACACTTTGTCC |

Quantitative RT–PCR primers for pluropotent genes and neural progenitor maker gene

| **Gene** | **Forward primer (5'-3')** | **Reverse primer (5'-3')** |
| --- | --- | --- |
| *OCT4* | CCTCACTTCACTGCACTGTA | CAGGTTTTCTTTCCCTAGCT |
| *NANOG* | CCTGTGATTTGTGGGCCTG | GACAGTCTCCGTGTGAGGCAT |
| *SOX2* | TGGCGAACCATCTCTGTGGT | CCAACGGTGTCAACCTGCAT |

Quantitative RT–PCR primer for transgenes

| **Gene** | **Forward primer (5'-3')** | **Reverse primer (5'-3')** |
| --- | --- | --- |
| *SF-hOCT4* | AAAAGAGCTCACAACCCCTC | CTTGGAAGCTTAGCCAGGTC |
| *SF-hLHX3* | AGGTAGACCACGCTCAGTT | AAGGCATTAAAGCAGCGTATCCA |
