# Supplementary material for: Sequentially induced motor neurons from human fibroblasts facilitate locomotor recovery in a rodent spinal cord injury model
Source: eLife. 2020 Jun 23;9:e52069. doi: 10.7554/eLife.52069 (PMC7311175; doi:10.7554/eLife.52069)
Supplement: Supplementary file 3. [file elife-52069-supp3.docx]

**Supplementary file 3. Summary of iMNIC induction from human fibroblast lines.**

| Fibroblast line | Gender | Age | Cell type | cells seeded per well | No. of colonies per well | Conversion efficiency to iMNICs | No. of established clones |
| --- | --- | --- | --- | --- | --- | --- | --- |
| HF1 | Female | 50 | Adult dermal | 5,000 | 9.7±1.6 | 0.2±0.2% | 6 |
| HF2 | Male | 68 | Adult dermal | 3,000 | 11.0±1.4 | 0.4±0.3% | 5 |
