# Supplementary material for: Sequentially induced motor neurons from human fibroblasts facilitate locomotor recovery in a rodent spinal cord injury model
Source: eLife. 2020 Jun 23;9:e52069. doi: 10.7554/eLife.52069 (PMC7311175; doi:10.7554/eLife.52069)
Supplement: Supplementary file 4. [file elife-52069-supp4.docx]

**Supplementary file 4. Characterization of established clones.**

| Clones | Parental cells | Proliferation capacity | Expression of ISL1 | Conversion efficiency to iMNs | RNA-Seq |
| --- | --- | --- | --- | --- | --- |
| HF1-iMNIC2 | HF1 | P15~ | + | *** | √ |
| HF1-iMNIC5 |  | P18~ | + | *** |  |
| HF1-iMNIC6 |  | P10~ | + | ** |  |
| HF1-iMNIC7 |  | P10~ | + | ** |  |
| HF1-iMNIC11 |  | P10~ | + | * |  |
| HF1-iMNIC12 |  | P10~ | + | *** |  |
| HF1-iMNIC1 | HF2 | P10~ | + | ** |  |
| HF2-iMNIC2 |  | P10~ | + | * |  |
| HF2-iMNIC4 |  | P10~ | + | ** |  |
| HF2-iMNIC6 |  | P16~ | + | *** | √ |
| HF2-iMNIC8 |  | P12~ | + | *** |  |

The gene expression of ISL1 was analyzed by qRT-PCR. “+” means positive expression.

Yields of HB9+iMN is represented by “*” (~70%). “**” (~80%), and “***” (~90%).
