# Supplementary material for: Sequentially induced motor neurons from human fibroblasts facilitate locomotor recovery in a rodent spinal cord injury model
Source: eLife. 2020 Jun 23;9:e52069. doi: 10.7554/eLife.52069 (PMC7311175; doi:10.7554/eLife.52069)
Supplement: Supplementary file 6. [file elife-52069-supp6.docx]

**Code for alignment and obtained alignment rates**

==============================================================================================

10_S250_L004_R1.fastq.gz

10_S250_L004_R2.fastq.gz

Aligning with HISAT2 sample 10_S250_L004

hisat2 -p 35 --dta-cufflinks -x /IndexedGenomesHisat/hg38_tran/genome_tran -1 /data/10_S250_L004_R1.fastq.gz -2 /data/10_S250_L004_R2.fastq.gz -S 10_S250_L004.sam

56433063 reads; of these:

56433063 (100.00%) were paired; of these:

6386886 (11.32%) aligned concordantly 0 times

42841687 (75.92%) aligned concordantly exactly 1 time

7204490 (12.77%) aligned concordantly >1 times

----

6386886 pairs aligned concordantly 0 times; of these:

2109648 (33.03%) aligned discordantly 1 time

----

4277238 pairs aligned 0 times concordantly or discordantly; of these:

8554476 mates make up the pairs; of these:

5119999 (59.85%) aligned 0 times

2223511 (25.99%) aligned exactly 1 time

1210966 (14.16%) aligned >1 times

95.46% overall alignment rate

11_S251_L004_R1.fastq.gz

11_S251_L004_R2.fastq.gz

hisat2 -p 35 --dta-cufflinks -x /IndexedGenomesHisat/hg38_tran/genome_tran -1 /data/11_S251_L004_R1.fastq.gz -2 /data/11_S251_L004_R2.fastq.gz -S 11_S251_L004.sam

59478851 reads; of these:

59478851 (100.00%) were paired; of these:

5879118 (9.88%) aligned concordantly 0 times

45391240 (76.31%) aligned concordantly exactly 1 time

8208493 (13.80%) aligned concordantly >1 times

----

5879118 pairs aligned concordantly 0 times; of these:

2083753 (35.44%) aligned discordantly 1 time

----

3795365 pairs aligned 0 times concordantly or discordantly; of these:

7590730 mates make up the pairs; of these:

4837559 (63.73%) aligned 0 times

1660178 (21.87%) aligned exactly 1 time

1092993 (14.40%) aligned >1 times

95.93% overall alignment rate

13_S252_L004_R1.fastq.gz

13_S252_L004_R2.fastq.gz

hisat2 -p 35 --dta-cufflinks -x /IndexedGenomesHisat/hg38_tran/genome_tran -1 /data/13_S252_L004_R1.fastq.gz -2 /data/13_S252_L004_R2.fastq.gz -S 13_S252_L004.sam

33962651 reads; of these:

33962651 (100.00%) were paired; of these:

3286733 (9.68%) aligned concordantly 0 times

26192074 (77.12%) aligned concordantly exactly 1 time

4483844 (13.20%) aligned concordantly >1 times

----

3286733 pairs aligned concordantly 0 times; of these:

1172770 (35.68%) aligned discordantly 1 time

----

2113963 pairs aligned 0 times concordantly or discordantly; of these:

4227926 mates make up the pairs; of these:

2824018 (66.79%) aligned 0 times

823979 (19.49%) aligned exactly 1 time

579929 (13.72%) aligned >1 times

95.84% overall alignment rate

3_S246_L004_R1.fastq.gz

3_S246_L004_R2.fastq.gz

hisat2 -p 35 --dta-cufflinks -x /IndexedGenomesHisat/hg38_tran/genome_tran -1 /data/3_S246_L004_R1.fastq.gz -2 /data/3_S246_L004_R2.fastq.gz -S 3_S246_L004.sam >>

63940404 reads; of these:

63940404 (100.00%) were paired; of these:

7715457 (12.07%) aligned concordantly 0 times

45754804 (71.56%) aligned concordantly exactly 1 time

10470143 (16.37%) aligned concordantly >1 times

----

7715457 pairs aligned concordantly 0 times; of these:

2164409 (28.05%) aligned discordantly 1 time

----

5551048 pairs aligned 0 times concordantly or discordantly; of these:

11102096 mates make up the pairs; of these:

7368161 (66.37%) aligned 0 times

2029616 (18.28%) aligned exactly 1 time

1704319 (15.35%) aligned >1 times

94.24% overall alignment rate

4_S247_L004_R1.fastq.gz

4_S247_L004_R2.fastq.gz

hisat2 -p 35 --dta-cufflinks -x /IndexedGenomesHisat/hg38_tran/genome_tran -1 /data/4_S247_L004_R1.fastq.gz -2 /data/4_S247_L004_R2.fastq.gz -S 4_S247_L004.sam

63796481 reads; of these:

63796481 (100.00%) were paired; of these:

7967789 (12.49%) aligned concordantly 0 times

46010159 (72.12%) aligned concordantly exactly 1 time

9818533 (15.39%) aligned concordantly >1 times

----

7967789 pairs aligned concordantly 0 times; of these:

2277931 (28.59%) aligned discordantly 1 time

----

5689858 pairs aligned 0 times concordantly or discordantly; of these:

11379716 mates make up the pairs; of these:

7569764 (66.52%) aligned 0 times

2063537 (18.13%) aligned exactly 1 time

1746415 (15.35%) aligned >1 times

94.07% overall alignment rate

5_S248_L004_R1.fastq.gz

5_S248_L004_R2.fastq.gz

hisat2 -p 35 --dta-cufflinks -x /IndexedGenomesHisat/hg38_tran/genome_tran -1 /data/5_S248_L004_R1.fastq.gz -2 /data/5_S248_L004_R2.fastq.gz -S 5_S248_L004.sam

72329430 reads; of these:

72329430 (100.00%) were paired; of these:

8208543 (11.35%) aligned concordantly 0 times

52901434 (73.14%) aligned concordantly exactly 1 time

11219453 (15.51%) aligned concordantly >1 times

----

8208543 pairs aligned concordantly 0 times; of these:

2417971 (29.46%) aligned discordantly 1 time

----

5790572 pairs aligned 0 times concordantly or discordantly; of these:

11581144 mates make up the pairs; of these:

7943478 (68.59%) aligned 0 times

2052220 (17.72%) aligned exactly 1 time

1585446 (13.69%) aligned >1 times

94.51% overall alignment rate

6_S249_L004_R1.fastq.gz

6_S249_L004_R2.fastq.gz

hisat2 -p 35 --dta-cufflinks -x /IndexedGenomesHisat/hg38_tran/genome_tran -1 /data/6_S249_L004_R1.fastq.gz -2 /data/6_S249_L004_R2.fastq.gz -S 6_S249_L004.sam

57540831 reads; of these:

57540831 (100.00%) were paired; of these:

6474361 (11.25%) aligned concordantly 0 times

42192855 (73.33%) aligned concordantly exactly 1 time

8873615 (15.42%) aligned concordantly >1 times

----

6474361 pairs aligned concordantly 0 times; of these:

2095618 (32.37%) aligned discordantly 1 time

----

4378743 pairs aligned 0 times concordantly or discordantly; of these:

8757486 mates make up the pairs; of these:

5524286 (63.08%) aligned 0 times

1839508 (21.00%) aligned exactly 1 time

1393692 (15.91%) aligned >1 times

95.20% overall alignment rate
