# Supplementary material for: Sequentially induced motor neurons from human fibroblasts facilitate locomotor recovery in a rodent spinal cord injury model
Source: eLife. 2020 Jun 23;9:e52069. doi: 10.7554/eLife.52069 (PMC7311175; doi:10.7554/eLife.52069)
Supplement: Supplementary file 7. [file elife-52069-supp7.docx]

**Code for obtaining genes counts and obtained statistics**

================================================================================================

htseq-count -i gene_name -m union -s no 3_S246_L004.sam hg38_ucsc.annotated.gtf > PatCouTxt

__no_feature 8017381

__ambiguous 1894979

__too_low_aQual 1836940

__not_aligned 2661793

__alignment_not_unique 30791905

htseq-count -i gene_name -m union -s no 4_S247_L004.sam hg38_ucsc.annotated.gtf > PatCouTxt

__no_feature 8650707

__ambiguous 1910786

__too_low_aQual 1864054

__not_aligned 2758202

__alignment_not_unique 22915669

htseq-count -i gene_name -m union -s no 5_S248_L004.sam hg38_ucsc.annotated.gtf > PatCouTxt

__no_feature 10645995

__ambiguous 1833506

__too_low_aQual 1940857

__not_aligned 2903423

__alignment_not_unique 30076802

htseq-count -i gene_name -m union -s no 6_S249_L004.sam hg38_ucsc.annotated.gtf > PatCouTxt

__no_feature 7824162

__ambiguous 1859356

__too_low_aQual 1644652

__not_aligned 1860310

__alignment_not_unique 23816094

htseq-count -i gene_name -m union -s no 10_S250_L004.sam hg38_ucsc.annotated.gtf > PatCouTxt

__no_feature 5901553

__ambiguous 1545346

__too_low_aQual 2099779

__not_aligned 1432766

__alignment_not_unique 13815975

htseq-count -i gene_name -m union -s no 11_S251_L004.sam hg38_ucsc.annotated.gtf > PatCouTxt

__no_feature 4443268

__ambiguous 1636577

__too_low_aQual 1552418

__not_aligned 1587881

__alignment_not_unique 15577872

htseq-count -i gene_name -m union -s no 13_S252_L004.sam hg38_ucsc.annotated.gtf > PatCouTxt

__no_feature 5914111

__ambiguous 783529

__too_low_aQual 792339

__not_aligned 984482

__alignment_not_unique 8953275
