# Supplementary material for: Sequentially induced motor neurons from human fibroblasts facilitate locomotor recovery in a rodent spinal cord injury model
Source: eLife. 2020 Jun 23;9:e52069. doi: 10.7554/eLife.52069 (PMC7311175; doi:10.7554/eLife.52069)
Supplement: Transparent reporting form [file elife-52069-transrepform.docx]

***eLife’s* transparent reporting form**

We encourage authors to provide detailed information *within their submission* to facilitate the interpretation and replication of experiments. Authors can upload supporting documentation to indicate the use of appropriate reporting guidelines for health-related research (see [EQUATOR Network](http://www.equator-network.org/%20)), life science research (see the [BioSharing Information Resource](https://biosharing.org/)), or the [ARRIVE guidelines](http://www.plosbiology.org/article/info:doi/10.1371/journal.pbio.1000412) for reporting work involving animal research. Where applicable, authors should refer to any relevant reporting standards documents in this form.

If you have any questions, please consult our Journal Policies and/or contact us: [editorial@elifesciences.org](mailto:editorial@elifesciences.org).

**Sample-size estimation**

- You should state whether an appropriate sample size was computed when the study was being designed
- You should state the statistical method of sample size computation and any required assumptions
- If no explicit power analysis was used, you should describe how you decided what sample (replicate) size (number) to use

Please outline where this information can be found within the submission (e.g., sections or figure legends), or explain why this information doesn’t apply to your submission:

In most experiments, we used triplicate experimental replicates at least for in vitro biological experiments which we believe acceptable. For in vivo animal experiments, sample size were more than 6-10 replicates per experiments. The information is provided in Materials and methods section.

**Replicates**

- You should report how often each experiment was performed
- You should include a definition of biological versus technical replication
- The data obtained should be provided and sufficient information should be provided to indicate the number of independent biological and/or technical replicates
- If you encountered any outliers, you should describe how these were handled
- Criteria for exclusion/inclusion of data should be clearly stated
- High-throughput sequence data should be uploaded before submission, with a private link for reviewers provided (these are available from both GEO and ArrayExpress)

Please outline where this information can be found within the submission (e.g., sections or figure legends), or explain why this information doesn’t apply to your submission:

Replicates (n) for each experiment is presented in corresponding figure legend as well as result section.

RNA-sequencing data have been deposited in NCBI's Gene Expression Omnibus and are accessible through GEO Series accession number GSE149664 (https://www.ncbi.nlm.nih.gov/geo/query/acc.cgi?acc=GSE149664).

Access token: kxuhqqwetrsrvgx (This token allows anonymous until publication)

**Statistical reporting**

- Statistical analysis methods should be described and justified
- Raw data should be presented in figures whenever informative to do so (typically when N per group is less than 10)
- For each experiment, you should identify the statistical tests used, exact values of N, definitions of center, methods of multiple test correction, and dispersion and precision measures (e.g., mean, median, SD, SEM, confidence intervals; and, for the major substantive results, a measure of effect size (e.g., Pearson's r, Cohen's d)
- Report exact p-values wherever possible alongside the summary statistics and 95% confidence intervals. These should be reported for all key questions and not only when the p-value is less than 0.05.

Please outline where this information can be found within the submission (e.g., sections or figure legends), or explain why this information doesn’t apply to your submission:

Statistical analysis methods are described in Materials and Methods section as well as figure legends. P-values, and error bars are presented for statistical test and can be found in the figure legends.

(For large datasets, or papers with a very large number of statistical tests, you may upload a single table file with tests, Ns, etc., with reference to sections in the manuscript.)

**Group allocation**

- Indicate how samples were allocated into experimental groups (in the case of clinical studies, please specify allocation to treatment method); if randomization was used, please also state if restricted randomization was applied
- Indicate if masking was used during group allocation, data collection and/or data analysis

Please outline where this information can be found within the submission (e.g., sections or figure legends), or explain why this information doesn’t apply to your submission:

We conducted blinded test for animal behavior test and described in Materials and methods section.

**Additional data files (“source data”)**

- We encourage you to upload relevant additional data files, such as numerical data that are represented as a graph in a figure, or as a summary table
- Where provided, these should be in the most useful format, and they can be uploaded as “Source data” files linked to a main figure or table
- Include model definition files including the full list of parameters used
- Include code used for data analysis (e.g., R, MatLab)
- Avoid stating that data files are “available upon request”

Please indicate the figures or tables for which source data files have been provided:

-For RNA-sequencing data, source and processing steps can be found at GEO (see above).

-Source data files have been provided for Figure 1, 2, and 5.

-Matlab software was used for RNA-sequencing data analysis and mentioned in materials and methods section (Transcriptomics global analysis and Gene-coverage count track plots)

-Source data files containing code used for our analysis were uploaded as separate files, ‘RNA seq data sequence summary’, ‘Code for alignment and obtained alignment rates’, and ‘Code for obtaining genes counts and obtained statistics’.
